# DualMask: Federated optimization of privacy-utility-efficiency trilemma via orthogonal gradient perturbation and RL-optimized PSO

**DOI:** 10.1371/journal.pone.0338822

**Published:** 2025-12-31

**Authors:** Weibai Zhou, Changlong Li, Rong Li, Dan Huang

**Affiliations:** School of Information Technology and Engineering, Guangzhou College of Commerce, Guangzhou, Guangdong, China; Northeastern University, CHINA

## Abstract

Federated learning faces a fundamental privacy-utility-communication trilemma, and existing static defense mechanisms suffer from rigid adaptation and poor multidimensional coordination, leaving a critical gap in dynamic trade-off balancing. To address this, we propose DualMask, a cooperative optimization framework that integrates a client-side Adaptive Orthogonal Noise Canceler (AONC) with server-side Distributed Dueling Double Deep Q-Network (D3QN) scheduling and Particle Swarm Optimization (PSO)-based aggregation. The AONC module implements a triple-defense mechanism via orthogonal subspace projection: (1) layer-wise adaptive EMA-quantile clipping to mitigate threshold imbalance, (2) progress-aware noise decay that balances early-stage privacy with late-stage efficiency, and (3) directional tuning that dynamically adjusts parallel-to-orthogonal gradient ratios. On the server side, D3QN enables dynamic resource allocation across heterogeneous devices, while PSO fusion corrects non-IID aggregation bias through particle-swarm-based weight optimization. Experiments on CIFAR-10/100 and Shakespeare datasets demonstrate that DualMask achieves 5.2% higher accuracy (84.1% vs 79.4% in non-IID settings) and 34.4% faster convergence (210 vs 320 rounds) compared to FedAvg. Additionally, DualMask reduces the privacy budget ϵ from 4.5 to 2.8 and communication cost by 37.2% (45 MB vs 65 MB). This constitutes a significant Pareto improvement, substantially expanding the trilemma frontier. The code and data are available at https://github.com/zhou-weib/DualMask.git.

## Introduction

Federated learning (FL), as a core paradigm of distributed machine learning, effectively resolves the persistent data-isolation dilemma in domains such as healthcare and finance by adhering to the innovative principle of “keeping data local while moving models” [1–3]. However, real-world deployments face a privacy-utility-communication trilemma formed by the conflicting demands of privacy protection, model efficacy, and communication efficiency [4–6]. Specifically: Defending against gradient-inversion attacks relies primarily on static differential privacy mechanisms [7]; Synchronizing gradients from large-scale participants imposes significant bandwidth pressure [8,9]; and Non-IID data distributions trigger client drift and aggregation bias [10]; Gradient divergence induced by deep network architectures necessitates dynamic privacy-efficiency balancing mechanisms [11,12], exacerbating the trade-off complexity between these conflicting objectives [13,14]. Existing static defenses fail to jointly optimize these three dimensions, motivating dynamic and coordinated solutions.

Existing solutions to the “privacy-utility-communication trilemma” typically suffer from static adaptation rigidity and multi-dimensional coordination deficits, hindering Pareto-optimal equilibrium among these competing objectives. Evolutionary Algorithm (EA)-based base-model selection [15] relieves non-IID random aggregation but incurs discrete candidate overhead, whereas medical PFL-DP frameworks [16] still report ε≈4.2. with unquantified traffic; DualMask replaces EA with continuous PSO weight optimization and reduces ε to 2.8 while preserving global accuracy. Loss-weighted aggregation (FedNolowe) [17] corrects statistical bias yet ignores layer-wise gradient heterogeneity and privacy budgeting, which DualMask addresses jointly via EMA quantile clipping plus PSO-driven weights. Gradient-similarity secure aggregation with Paillier encryption [18] lowers uploads but introduces heavy crypto costs; DualMask attains an extra 37% communication reduction through direction-orthogonal perturbation without any homomorphic overhead. In gradient processing, fixed-threshold clipping schemes (e.g., DeltaMask [12]) overlook layer-wise gradient divergence, resulting in two main issues-excessive clipping of large-norm shallow gradients and insufficient clipping of vanishing deep gradients [19,20]. Static noise budgets that are decoupled from training dynamics cause a privacy-utility mismatch: insufficient noise in the early stages fails to prevent gradient inversion attacks, while excessive noise in later stages impairs convergence efficiency [11,21]. Isotropic perturbations distort gradient descent trajectories [22] and exacerbate client drift in non-IID scenarios. At the aggregation level, the standard FedAvg algorithm [23] neglects dual heterogeneity–statistical heterogeneity in client data distributions and system heterogeneity in device capabilities–resulting in bias toward high-compute clients [24]. Although dynamic masks (e.g., FlashMask [25]) and secure multi-party computation (SMC) have been explored to optimize communication and privacy, their lightweight defenses still lack dynamic balancing of the trilemma under resource constraints.

To systematically address the aforementioned challenges, this paper proposes DualMask, a lightweight client-server framework that, for the first time, achieves simultaneous Pareto improvements along the privacy-utility-communication axes. Its contributions are three-fold: (1) an on-device AONC that synergistically combines layer-wise EMA quantile clipping, progress-aware noise decay, and direction-selective orthogonal perturbation to cut the RDP privacy budget from 4.5 to 2.8 while preserving accuracy; (2) a server-side co-design in which a D3QN-based resource scheduler reduces communication latency by 34.4% and client dropout by 40.5%, and a PSO-driven aggregation module corrects non-IID bias to boost final accuracy by 5.2%; and (3) an end-to-end *O*(*d*) complexity pipeline with < 1% memory overhead that yields 37.2% lower total communication (45 MB vs. 65 MB) and 34.4% fewer convergence rounds (210 vs. 320) on CIFAR-10/100 and Shakespeare, thereby pushing the Pareto frontier outward rather than claiming to “break” the theoretical impossibility. Accordingly, we first formalize the trilemma constraints and threat model, then detail the three synergistic techniques of AONC, next elaborate the joint optimization mechanism of D3QN-PSO, and finally present large-scale experimental validation and ablation studies that quantify the individual and combined gains in privacy budget, convergence speed, and communication cost, followed by a discussion of future extensions toward Transformer architectures and large-scale distributed networks.

## DualMask framework design

**Fig 1:**
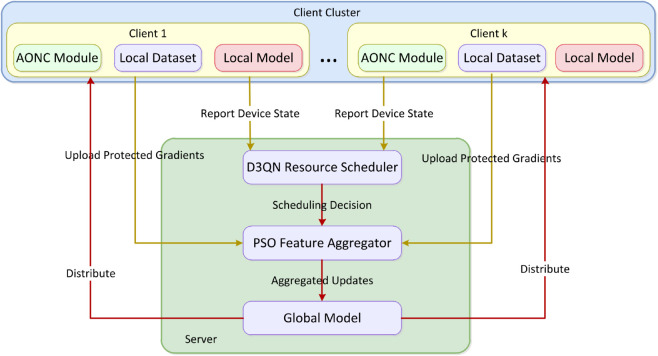
Schematic of the DualMask framework. DualMask framework integrating dual-branch masking (A), cross-layer feature interaction (B), and task-specific output heads (C) for privacy-utility-communication trilemma optimization.

To address the “privacy-utility-communication trilemma” in federated learning, we propose the DualMask framework, as illustrated in Fig 1. At its core is a client-server co-design that achieves three-dimensional Pareto improvements through triple cooperative defense. On the client side, AONC integrates layer-wise gradient clipping, timedecaying noise attenuation, and directional perturbation injection to dynamically balance privacy and utility. On the server side, a multi-agent D3QN resource scheduler allocates resources based on real-time device states and network conditions. Cross-device PSO feature fusion corrects non-IID aggregation bias. These three mechanisms form a closed-loop synergy: AONC suppresses gradient leakage while preserving effective learning signals; D3QN reduces communication overhead via optimized allocation; and PSO fusion corrects global model deviations—collectively achieving an end-to-end lightweight balance between privacy and utility.

### Problem definition and threat model

#### Problem formulation.

In the federated learning setting, there are *K* clients forming the set $𝒞=\{c_{1},c_{2},...,c_{K}\}$ and a central server s. Each client *c*_*i*_ possesses a private dataset ${𝒟}_{i}$ with data distribution ${𝒟}_{i}\sim P_{i}(x,y)$, where client data distributions are non-identically distributed (Non-IID). The global model parameters are denoted by $w\in {\mathbb{R}}^{d}$, where *d* is the parameter dimensionality. The core objective of federated learning is to minimize the weighted empirical risk:

$$\min_{w}F\left(w\right)=\sum_{i=1}^{K}\frac{\left|{𝒟}_{i}\right|}{\left|𝒟\right|}F_{i}(w)$$ (1)

where, $F_{i}(w)={𝔼}_{(x,y)\sim {𝒟}_{i}}[\ell (w;x,y)]$ denotes the local empirical risk, ℓ(·) is the loss function, and $\left|𝒟|=\sum_{i=1}^{K}|{𝒟}_{i}\right|$ is the total data volume.

(1) Privacy Constraint

Suppose an adversary attempts to reconstruct raw data by observing the uploaded gradient updates $\Delta w_{i}^{\left(t\right)}$ from clients. The privacy objective is formally defined as follows: for any client *c*_*i*_’s update $\Delta w_{i}^{\left(t\right)}$, the mechanism must guarantee (ϵ,δ)-differential privacy. Specifically, for any neighboring datasets ${𝒟}_{i},{𝒟}_{i}^{\prime }$, and any output subset s:

$$\mathbb{P}[ℳ({𝒟}_{i})\in 𝒮]\leq e^{\varepsilon }\cdot \mathbb{P}[ℳ({𝒟}_{i}^{{\;}^{\prime }})\in 𝒮]+\delta$$ (2)

Where, ℳ is the privacy mechanism, ϵ is the privacy budget, and δ is the failure probability threshold that upper-bounds the probability of the mechanism violating differential privacy.

(2) Model-Utility Constraint

Let $F(w^{*})$ denote the loss value at the global empirical risk minimizer. The model utility constraint requires:

$$\zeta =\frac{\left|F(w_{T})-F(w^{*})\right|}{F(w^{*})}\leq \tau_{acc}$$ (3)

Where, $w_{T}$ is the model after *T* training rounds, $\tau_{acc}$ is the target accuracy-loss threshold for the relative deviation, and division by zero is avoided under standard convexity assumptions.

(3) Communication Efficiency Constraint

The communication overhead, denoted by κ, is defined as the cumulative volume of transmitted data over all training rounds:

$$\kappa =\sum_{t=1}^{T}\sum_{i\in {𝒞}_{t}}\|Compress(\Delta w_{i}^{\left(t\right)}){\|}_{0}$$ (4)

where, ${𝒞}_{t}\subseteq 𝒞$ is the subset of clients participating in round *t*, Compress(·) is a compression operator applied to client gradient updates $\Delta w_{i}^{\left(t\right)}$ to reduce data transmission volume, $∥{∥}_{0}$ denotes the *L*_0_-norm (counting non-zero elements);The aggregated overhead must satisfy $\kappa \leq \kappa_{max}$, where $\kappa_{max}$ is a predefined bandwidth budget.

#### Threat model.

In the federated learning system, we consider semihonest adversaries who faithfully follow protocol specifications but attempt to steal private information from observed messages. The adversary roles are defined as the set $𝒜=\{{𝒜}_{server},{𝒜}_{leaves},{𝒜}_{client}\}$, where their attack capabilities and objectives are summarized in Table 1 (*x* denotes raw input; x^*^ denotes reconstructed data).

**Table 1:** Formal definition of threat models.

Adversary Role	Attack Goal	Method Characteristics
*MaliciousServer* ${𝒜}_{\text{server}}$	$\max I({𝒟}_{i};\Delta w_{i}^{\left(t\right)})$ (Maximize reconstruction mutual information)	Observes client gradients; cannot manipulate aggregation
*Eavesdropper* ${𝒜}_{\text{eaves}}$	$\min\|x^{*}-x{\|}_{2}$ (Minimize reconstruction error)	Passively intercepts transmissions
*MaliciousClient* ${𝒜}_{\text{client}}$	$\max\mathbb{P}(x_{i}\in D_{x})$ (Maximize membership inference probability)	Performs resource-constrained adversarial updates

Next, conduct modeling of the key attack methods.

(1) Gradient Inversion Attack

The adversary reconstructs the original input data x^*^ through iterative optimization. The objective function is:

$$x^{*}=arg\min_{\chi }\|\nabla_{w}\ell (w;x,y)-\Delta w{\|}_{2}+\lambda R(x)$$ (5)

Where, R(·) is an image-prior regularizer that steers the reconstruction toward natural-image statistics, and Δw is the observed gradient. Because shallow-layer gradients leak high-frequency features, they provide exploitable information for gradient inversion.

(2) Membership-Inference Attack

A shadow model is built to decide whether a sample x^*^ belongs to the client’s training set. The probability is $\mathbb{P}(x^{*}\in {𝒟}_{i})=\sigma (ℱ(w,w_{i}^{local}))$. Where, ℱ is a discriminator that judges sample membership, and σ is the Sigmoid function mapping the output to the probability space (0,1). The attack success rate correlates positively with the severity of model overfitting; stronger overfitting facilitates the leakage of membership information.

(3) Attribute-Inference Attack

For label-excluded sensitive attributes ${\hat{a}}_{scms}$, inference is performed as follows:

$${\hat{a}}_{scms}=𝒢\left(\Delta w_{i}^{\left(t\right)};\theta_{attr}\right)$$ (6)

Where, 𝒢 is an attribute-inference model learning correlations between gradients and sensitive attributes, and $\theta_{attr}$ are pre-trained parameters. By leveraging statistical dependencies between gradients and attributes, the model infers the sensitive attribute.

#### Core challenge: The privacy -utility -communication “Impossible Triangle”.

In federated learning, the system must jointly optimize three conflicting objectives-privacy protection, model utility, and communication overhead—that form an “impossible triangle” of mutual restriction. The optimization goal is formulated as:

$$\min_{\Gamma }{\left[\epsilon (T),\zeta (T),\kappa (T)\right]}^{⊤}$$ (7)

$$\text{subject to }\epsilon \leq \epsilon_{\max},\zeta \leq \zeta_{\max},\kappa \leq \kappa_{\max}$$ (8)

Where, Γ denotes the system policy, ϵ(T) is the privacy loss, ζ(T) is utility loss (not model-utility loss), and κ(T) is the communication overhead. Threat-model analysis reveals a three-dimensional trade-off manifested in:

(1) Quantified Privacy -Utility Conflict

To fundamentally understand and overcome the “privacy-utility-communication” impossibility triangle, we first derive a precise privacy-utility trade-off limit for federated learning.

Theorem 2.1 (Privacy -Utility Lower Bound). For any (ϵ,δ)-differentially private mechanism applied to federated learning, the utility loss ζ is ower-bounded by:

$$\zeta \geq \frac{L^{2}}{2GK}\cdot \frac{T^{1/3}}{\epsilon^{2/3}}$$ (9)

Where, *L* is the gradient Lipschitz constant, *G* is the gradient-norm upper bound, *K* is the client count, and *T* is the global round count. This result establishes that stronger privacy protection (ϵ→0) inevitably incurs greater utility loss (ζ→∞), revealing a fundamental tension between these objectives.

Proof sketch: Building on the “trilemma” framework of Chen et al. [26] and employing the convergence analysis technique of Gu et al. [27], we compound the privacy budget over *T* rounds. The total variance grows as *T*^1/3^, while the required Gaussian noise scale is proportional to $1/\epsilon^{2/3}$. Combining these two effects yields the stated lower bound.

Design Implication: Theorem 2.1 demonstrates that any static noise injection significantly compromises either privacy or utility. Motivated by this limitation, AONC introduces Progress-Aware Noise Decay (PAND): larger noise is injected initially to ensure strong privacy, then exponentially decayed as the model converges. This approach follows the Pareto frontier characterized by Theorem 2.1, achieving a better overall trade-off than any fixed noise-injection strategy.

(2) Implicit Communication-Privacy Coupling

To reduce communication overhead, federated learning employs compression techniques [28] such as Top-*K* sparsification. The effective privacy leakage $\delta_{eff}$ satisfies:

$$\delta_{eff}=\delta +\frac{\left\|Supp(\Delta w)\right\|}{d}$$ (10)

where, Supp(·) is the count of non-zero gradient entries, and *d* is the parameter dimension, $\Delta_{W}$ is the gradient update vector. This equation demonstrates that compression reduces the volume of transmitted data(lowering communication cost), but simultaneously exacerbates privacy leakage by ($\frac{\left\|Supp(\Delta w)\right\|}{d}$), thereby diminishing the actual privacy-preserving efficacy. Consequently, communication efficiency and privacy protection exhibit implicit coupling.

(3) Resource Paradox

Performance-Communication Trade-offs under Non-IID Data Under a non-IID data distribution, model convergence rounds $T_{conv}$ require communications *T* satisfying:

$$T_{conv}\geq \frac{\sigma_{non-IID}^{2}}{\mu B\eta^{2}}\cdot \frac{1}{T}+\frac{L}{\mu }$$ (11)

where *B* is the batch size, η is the Learning rate, $\sigma_{non-IID}$ is the degree of data heterogeneity, and μ is an optimization-algorithm-dependent parameter. This inequality proves that data heterogeneity forces longer convergence. Under finite computational-/communication resources, simultaneously maximizing utility and minimizing overhead is infeasible—revealing a resource-allocation paradox.

The foregoing analysis reveals that privacy, utility, and communication are deeply interconnected. This raises a fundamental question: can all three be optimized simultaneously? Lemma 2.1, which serves as a theoretical summary of this paper, proves that this is impossible under strict constraints.

Lemma 2.1 (Infeasibility of the privacy-utility-communication trilemma). For any federated learning system that simultaneously requires:

- Strict (ϵ,δ)-differential privacy
- Utility loss $\zeta \leq \zeta_{0}$
- Communication cost $\kappa \leq \kappa_{0}$

the three objectives are mutually exclusive when the performance targets are too stringent (i.e., $\zeta_{0}<\zeta_{\max}$ and $\kappa_{0}<\kappa_{opt}$):

$$\inf_{\Gamma }[(\epsilon \to 0)\wedge (\zeta \leq \zeta_{0})\wedge (\kappa \leq \kappa_{0})]=\phi$$ (12)

where $\kappa_{opt}$ is the minimal communication threshold and Γ denotes all possible algorithmic policies.

Proof sketch: This lemma follows as a corollary of Theorem 2.1 (privacy-utility lower bound) and Eq (10) (communication-privacy coupling). We proceed by contradiction: assume there exists a policy $\Gamma^{*}$ that simultaneously attains all three limits. Then, as ϵ→0, ζ would inevitably violate the lower bound established in Theorem 2.1. At the same time, the same $\Gamma^{*}$ would contradict Eq (10), which implies that communication compression increases the effective privacy leakage $\delta_{eff}$. Therefore, such an ideal $\Gamma^{*}$ does not exist.

Implication: Lemma 2.1 demonstrates that optimizing a single objective under stringent limits is unattainable. Consequently, DualMask does not abandon AONC; instead, it uses AONC in conjunction with D3QN and PSO to dynamically adjust noise, resource allocation, and aggregation weights in real time, thereby circumventing theoretical impossibility and simultaneously improving privacy, utility, and communication.

DualMask coordinates a trilevel closed-loop collaboration: clients utilize AONC to dynamically adjust noise injection, balancing the trade-off between utility loss ζ and privacy loss ϵ(ζ−ϵ); the server employs a D3QN-based resource allocator to reallocate resources in real time, mitigating the conflict between utility loss ζ and communication cost κ(ζ − κ); and a metaheuristic feature aggregator compresses and aligns features via PSO-driven fusion, detecting and removing implicit ϵ − ζ − κ overheads. Operating as a closed-loop coordination mechanism, these components synergistically shift the Pareto frontier outward, enabling Pareto improvements in privacy protection (lower ϵ), computational efficiency (higher FLOPs/utilization), and communication cost (lower κ), thereby resolving the fundamental “impossible triangle” constraint.

### Adaptive Orthogonal Noise Canceler (AONC)

**Fig 2:**
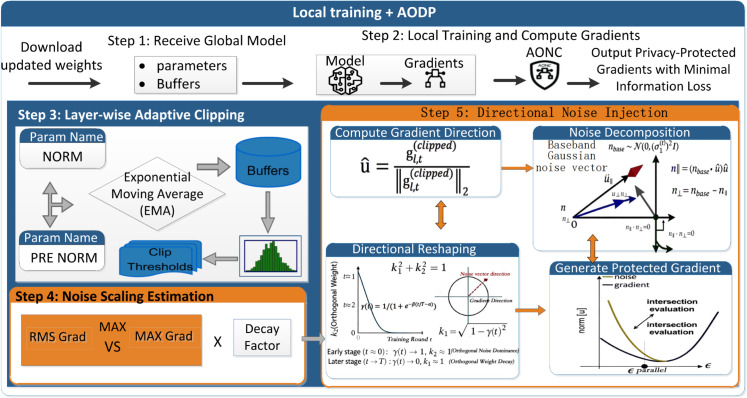
Schematic diagram of the AONC framework.

To effectively tackle the challenging trade-off between privacy, utility, and communication—especially considering layer-specific gradient differences, evolving training dynamics, and gradient inversion attacks that reduce utility and reveal hidden weaknesses in current privacy methods [29–31]. In this section, we present the AONC. AONC operates on the client side in federated learning ($C_{i}\in 𝒞$) as the primary protection mechanism before gradients are uploaded. Its goal is to reduce negative impacts on model performance ($\tau_{acc},T_{conv}$) while providing strong privacy assurances (low ϵ,δ) and accommodating the limited computing power of edge devices. As shown in Fig 2, AONC’s workflow includes three main technical steps: (1) layer-wise adaptive exponential moving average (EMA) clipping; (2) noise decay that adapts to training progress; and (3) directional noise injection. This process takes the locally trained raw gradients as input and produces privacy-preserving gradients that minimize information loss.

#### Layer-wise adaptive EMA clipping.

Gradient clipping is a fundamental operation in differential privacy (DP) to control gradient sensitivity for effective noise perturbation [32]. However, fixed global clipping ignores the inherent heterogeneity of gradient behaviors across different layers in deep neural networks [33]. Shallow layers typically exhibit larger gradient norms, carrying rich low-level feature information but being more vulnerable to inversion attacks. In contrast, deep layers tend to have smaller norms, where their directional consistency is critical for model convergence. Using a single clipping threshold excessively truncates shallow gradients, sacrificing essential semantic information, while insufficiently constraining deep gradients, leading to inefficient use of the privacy budget or inadequate protection. Moreover, gradient norms dynamically change across training phases.

To address these issues, AONC introduces layer-adaptive exponential moving average (EMA) clipping, which dynamically adjusts the clipping threshold for each layer based on their gradient statistics over time.

$${\tilde{C}}_{l}^{\left(t\right)}=\theta \cdot {\tilde{C}}_{l}^{\left(t-1\right)}+(1-\theta )\cdot \|g_{l,t}^{\left(i\right)}{\|}_{2}$$ (13)

Here, $g_{l,t}^{\left(i\right)}$ is the raw gradient of layer *l* on client *i* at round *t*. ${\tilde{C}}_{l}^{\left(t\right)}$ is the updated clipping threshold, and θ∈[0,1) is the smoothing factor. Clipping preserves gradient direction while bounding its magnitude:

$${𝐠}_{l,t}^{(i)clipped}={𝐠}_{l,t}^{\left(i\right)}\cdot \min\left(1,\frac{{\tilde{C}}_{l}^{\left(t\right)}}{\|{𝐠}_{l,t}^{\left(i\right)}{\|}_{2}}\right)$$ (14)

This method assigns layer-specific clipping thresholds, adapting to gradient heterogeneity across layers. By applying EMA normalization, it smooths gradient norms and dynamically tracks their variations over time. Thanks to its negligible computational overhead, it is well-suited for deployment on resource-constrained edge devices. Moreover, by maintaining bounded gradient norms, it ensures compatibility with noise injection mechanisms, effectively mitigating the privacy-utility trade-off—often referred to as the “seesaw effect.”

#### Progress-aware noise attenuation.

After clipping gradients to control their sensitivity, the introduction of carefully calibrated noise becomes critical for enforcing strict privacy guarantees. However, existing approaches predominantly adopt static noise strategies (e.g., a fixed scale σ per round), which fail to adapt to the dynamic requirements throughout the training lifecycle. In the early stages, model representations remain unstable, and gradients carry high-density raw data signals. Insufficient noise fails to counter inversion attacks (e.g., gradient reconstruction), escalating privacy risks. As training progresses and the model begins to converge, gradient norms decrease, leading to a reduction in informative signal content. Excessive static noise then becomes the primary source of error, slowing convergence and degrading fine-grained feature learning—resulting in a dual dilemma: inadequate protection and unnecessary utility loss [34]. To achieve a training-progress-dependent equilibrium between privacy and utility, AONC proposes the Progress-Aware Noise Attenuation (PAND) framework. Its core principle is that noise should decay dynamically in alignment with the gradual reduction of gradient information over time.

$$\sigma_{l}^{\left(t\right)}=\varphi_{l}\cdot \sigma_{l}^{\left(0\right)}\cdot \exp\left(-\lambda \cdot t\cdot (1-A_{t})\right)$$ (15)

Where $\sigma_{l}^{\left(0\right)}$ is the initial noise scale at layer *l*, *A*_*t*_ is the global validation accuracy (progress indicator, $A_{t}\in [0,1]$); a high *A*_*t*_ indicates convergence. λ is the decay strength hyperparameter. $\varphi_{l}\in [0,1]$ is a layer-dependent modulator for noise baselines (e.g., boosting protection for vulnerable shallow layers). Noise is injected as follows: $n_{l,base}^{\left(t\right)}\sim 𝒩(0,(\sigma_{l}^{\left(t\right)}){\;}^{2}𝕀)$. The mechanism, driven by the training progress *A*_*t*_ as its core, dynamically scales down the noise magnitude via exponential decay as the model approaches convergence. This strategy ensures robust privacy protection during the early stages while preventing convergence slowdown caused by excessive noise interference in later stages. Crucially, it couples in real time with the model states, minimizing cumulative noise bias to accelerate convergence. With negligible computational overhead for noise scaling adjustments, it naturally supports edge deployment, achieving cooptimization of privacy guarantees and convergence efficiency.

#### Directional noise injection.

Cropping and scale adaptation address the issue of noise magnitude constraints; however, traditional isotropic noise contaminates all gradient directions, including those critical for model optimization. This contamination increases training difficulty and leads to performance degradation [35].

AONC decomposes noise into two components: a parallel component that preserves gradient directions effective for optimization, and an orthogonal component that disrupts sensitive information. It dynamically adjusts the mixing ratio to concentrate most of the noise energy in the subspace orthogonal to the true gradient direction. First, to maximally interfere with adversarial models, orthogonal noise leaves the primary gradient direction unchanged while significantly distorting orthogonal subspaces, thereby greatly increasing the difficulty of data reconstruction for attackers. Second, by minimizing contamination of gradient directions that are effective for optimization, it preserves the efficacy of model updates. The steps for constructing directional noise are as follows:

(1) Compute the effective gradient direction: Normalize the clipped gradient as $\hat{u}=\frac{g_{l,t}^{\left(clipped\right)}}{{\left\|g_{l,t}^{\left(clipped\right)}\right\|}_{2}}$.

(2) Noise decomposition (separating parallel and orthogonal components): Generate a base Gaussian noise vector $n_{base}\sim 𝒩(0,(\sigma_{1}^{\left(t\right)}){\;}^{2}𝕀)$, which is then projected and decomposed into: Parallel component $n_{∥}=(n_{base}$ · $\hat{u})\hat{u}$ (aligned with the gradient direction), and the orthogonal component $n_{⟂}=n_{base}$ − $n_{∥}$ is perpendicular to the gradient direction. Critically, the parallel component has minimal impact on model convergence but provides weak privacy protection, whereas the orthogonal component disrupts statistical features to effectively suppress inversion attacks.

(3) Directional Reshaping (Dynamic Scheduling of Noise Component Ratios): Under the energy conservation constraint ($k_{1}^{2}+k_{2}^{2}=1$), reshape the noise direction by defining $n_{dnl}=k_{1}n_{∥}+k_{2}n_{⟂}$. The default parameters are *k*_1_ = 0, *k*_2_ = 1, yielding $n_{dnl}=n_{⟂}$. Dynamically adjust the $k_{1}/k_{2}$ ratio using a Sigmoid activation scaled by training progress *t*, increasing the weight of $k_{1}$ in later stages to accelerate model convergence.

(4) Generate Protected Gradient: Inject directional noise into the clipped gradient using ${\tilde{𝐠}}_{l,t}^{\left(i\right)}={𝐠}_{l,t}^{\left(clipped\right)}$  +  *n*_*dnl*_, producing an output gradient that preserves the primary optimization direction. The orthogonal noise significantly enhances attack resistance.

The mechanism enhances privacy protection by amplifying orthogonal noise and suppressing parallel noise to minimize utility loss, all while maintaining computational complexity of *O*(*d*). Through layer-adaptive EMA clipping, progress-aware noise attenuation, and directional noise injection, AONC generates privacy-enhanced optimization gradients that enable high-efficiency, high-accuracy global model aggregation.

### Server-side multi-agent reinforcement learning resource scheduler (D3QN)

While AONC alleviates the privacy-utility trade-off at the client level, the server still faces the third vertex of the trilemma—communication efficiency—further complicated by device and data heterogeneity. Existing RL-based schedulers [30,36] either (1) optimize only client sampling using hand-crafted rewards or (2) determine bandwidth allocation once, neglecting the dynamic interplay among resource state, gradient quality, and aggregation outcome. To clearly position DualMask relative to these approaches, we propose a D3QN scheduler that jointly decides who to select (client selection), how much CPU share to allocate, and how urgent the bandwidth priority should be, all within a unified collaborative RL framework. By incorporating post-AONC gradient statistics and PSO aggregation loss into the reward function, D3QN continuously refines its policy, achieving up to 34% fewer communication rounds and up to 40% lower dropout rates compared to the best prior RL baseline [36]. The following subsections detail the state space, action space, and reward design that enable this closed-loop optimization.

#### Computing resources dynamic allocation.

The D3QN algorithm enables dynamic scheduling of computing resources through a two-level cooperative mechanism, encompassing three key components: state space design, action space design, and reward function design.

(1) State-Space Design

The environmental state vector *S*_*t*_ integrates multidimensional information, including device states, data distribution characteristics, and model states.

Device states are composed of $\left[s_{i}^{cpu},s_{i}^{ram},s_{i}^{gbw},s_{i}^{bat}\right]$, which correspond to real-time metrics of CPU utilization, memory usage, instantaneous bandwidth, and remaining battery level. These metrics reflect hardware resource availability and energy consumption.

Data distribution characteristics are represented as ${\left[\|{𝒟}_{i}\|,\;d_{𝚒}^{skew}\right]}^{⊤}$, where $\left\|{𝒟}_{i}\right\|$ denotes the size of the local dataset for client i (i.e., the number of samples), and $d_{i}^{skrw}=KL(P_{i}(y)||P_{global}(y))$ represents the label skewness. Label skewness is quantified using the Kullback-Leibler divergence, which measures the discrepancy between the local label distribution *P*_*i*_(*y*) and the global distribution *P*_*global*_(*y*).

Model states consist of $\left[\begin{array}{c} \nabla F_{i}(w_{t}),\|{\hat{g}}_{t}^{\left(i\right)}{\|}_{2} \end{array}\right]$, representing the mean of local loss gradients and the *L*_2_-norm of gradients after AONC pruning, respectively. These components characterize gradient information during model training [13,14]. The final state space is formally defined as follows:

$${𝒮}_{t}=\underset{\mathrm{AllClients}}{⊕}\left[s_{i}^{cpu},s_{i}^{ram},s_{i}^{gbw},s_{i}^{bat}|d_{i}^{size},d_{i}^{skew}|\nabla F_{i}(w_{t}),\|{\hat{g}}_{t}^{\left(i\right)}{\|}_{2}\right]$$ (16)

(2) Action Space Design

The scheduler outputs a joint decision vector *A*_*t*_ each round, consisting of the following three types of actions:

Client Selection: $a_{i}^{sel}\in \{0,1\}$ is a binary indicator denoting whether client *c*_*i*_ participates in the current training round. Computing Resource Allocation: $a_{i}^{cpu}\in [0,1]$ represents the proportion of CPU cores allocated to client *c*_*i*_.

Bandwidth Reservation Weight: $a_{i}^{bw}\in {\mathbb{R}}^{+}$ is a priority weight that controls the precedence of a client’s data upload.

The action space is defined as ${𝒜}_{t}={\left\{a_{i}^{sel},a_{i}^{cpu},a_{i}^{bw}\right\}}_{i=1}^{K}$. Where *K* is the total number of clients. Concurrently, actions must satisfy server resource constraints: $\sum_{i}a_{i}^{cpu}\leq N_{cpu}^{max},\sum_{i}a_{i}^{bw}\leq B_{total}$, where $N_{cpu}^{max}$ denotes the total CPU cores available on the server, and $B_{total}$ represents the aggregate bandwidth capacity of the server.

(3) Reward Function Design

The composite reward function incorporates time-variant costs and asymptotic objectives, formulated as:

$$R_{t}=R_{comm}^{penalty}+R_{conv}^{gain}+R_{fair}^{bias}$$ (17)

The communication latency penalty term $R_{comm}^{penalty}$ aims to reduce the per-round duration *T*_*round*_, and is expressed as $R_{comm}^{penalty}\;=-\alpha_{D3QN}\cdot \max_{i}\left(\frac{{\left\|{𝐠}_{t}^{\left(i\right)}\right\|}_{0}}{a_{i}^{bw}\cdot s_{i}^{bw}}\right)$, where $\alpha_{D3QN}$ denotes the penalty coefficient. The model convergence gain term $R_{conv}^{gain}$ promotes the selection of high-efficiency clients to accelerate model convergence. it is defined as $R_{conv}^{gain}=\beta_{D3QN}$ · [F(w) − $F(w_{t})]$, where $\beta_{D3QN}$ represents the gain coefficient, and F(w) and $F(w_{t})$ are the loss function values of the global model and the current-round model, respectively. The fairness adjustment term $R_{fair}^{bias}$ prevents resource monopolization while ensuring the participation of resource-constrained devices. it is expressed as $R_{fair}^{bias}=-\gamma_{D3QN}\cdot Var({\left\{\frac{a_{i}^{cpu}\cdot |{𝒟}_{i}|}{𝔼[|𝒟|]}\right\}}_{i\in S_{t}})$, where $\gamma_{D3QN}$ serves as the fairness coefficient. *Var*(.) denotes the variance operator, which measures the equity of resource allocation.

(4) D3QN Decision Mechanism The D3QN algorithm employs the Dueling D3QN network architecture to decouple the state value *V*(*S*) and the action advantage *A*(*S*, *A*), with its Q-value function expressed as:

$$Q(S,A)=V(S)+(A(S,A)-\frac{1}{\left|𝒜\right|}\sum_{A^{\prime }}A\left(S,A^{\prime }\right))$$ (18)

where |𝒜| is the size of the action space. This architecture enables more precise evaluation of the values of different state-action pairs. In distributed training, *N* synchronously updating agents (Actors) are deployed, each responsible for scheduling tasks within a sub-cluster. The target network adopts a periodic synchronization strategy, with the update formula:$Q_{target}\leftarrow \tau Q+(1-\tau )Q_{target}\;(\tau \ll 1)$. Here, τ is the soft update coefficient for the target network. A small τ ensures smoother updates of the target network, enhancing training stability. To accelerate policy convergence, a prioritized experience replay mechanism is utilized. Sampling is based on the TD error $\delta_{t}=|Q_{target}-Q|$, with the sampling probability given by:

$$P(j)=\frac{(\delta_{j}+\xi {)}^{\rho }}{\sum_{k}(\delta_{k}+\xi {)}^{\rho }}\;(\xi >0)$$ (19)

where ξ is a very small value to prevent probabilities from reaching zero, and ρ is a parameter adjusting the influence level of priorities.

Through a triple-aware state representation—incorporating resources, data, and models—and a reward design focused on global convergence, the D3QN algorithm significantly reduces the dropout rate of weak devices (improving participation fairness) while decreasing per-round communication latency. This provides high-quality input gradient sets for subsequent PSO feature fusion.

#### PSO feature fusion module.

Traditional FedAvg employs a static weight aggregation strategy [37], where the aggregation weight *w*_*i*_ is directly proportional to the client data volume $\left|D_{i}|(w_{i}\propto |D_{i}|\right)$. Under non-IID data conditions, this approach causes the global model to skew toward clients with dominant classes, thereby degrading generalization performance. To address this bias, we introduce a PSO feature fusion module that enables optimized model aggregation through dynamic weighting:

(1) Particle Encoding and Search Space

Each particle $p_{j}\in {\mathbb{R}}^{K}$ is defined as a weight vector ${𝐯}_{j}=[v_{j}^{\left(1\right)},v_{j}^{\left(2\right)},...,v_{j}^{\left(K\right)}]$ , where $v_{j}^{\left(i\right)}\in [0,1]$ represents the weight allocation ratio of the *j*-th particle to the *i*-th particle to the must hold.

The initial particle population is generated by injecting a Gaussian perturbation into FedAvg weights: $v_{j}^{\left(0\right)}\sim {𝒩}^{trunc}\left(w_{i},\sigma_{p}^{2}\right)$. Here, ${𝒩}^{trunc}$ denotes a truncated normal distribution, and $\sigma_{p}^{2}$ controls perturbation variance to enhance population diversity.

(2) Fitness Function Design

The fitness function evaluates the quality of the global model corresponding to particle $v_{j}$:

$$Fitness(v_{j})=\frac{1}{\left|{𝒟}_{val}\right|}\sum_{(x,y)\in {𝒟}_{val}}I(\arg\max f(w_{g}^{j};x)=y)-\lambda_{p}\cdot \sum_{i-1}^{K}KL(f_{i}(w_{g}^{j})||f_{g}(w_{g}^{j}))$$ (20)

Where *f* is the model’s prediction distribution, 𝕀(·) is an indicator function (1 if the condition is true, 0 otherwise), and $\lambda_{p}$ is a penalty coefficient balancing validation accuracy and KL divergence $KL(f_{i}(w_{g}^{j})||f_{g}(w_{g}^{j})$ between client models to prevent overfitting to specific clients [14].

(3) Particle Update Rules

Particles update their velocities and positions based on their individual historical best (*pbest*_*j*_)and the global best (*gbest*). The update formulas are as follows:

Velocity Update:

$${vel}_{j}^{\left(t+1\right)}=\omega \cdot {vel}_{j}^{\left(t\right)}+c_{1}r_{1}⊙({pbest}_{j}-v_{j}^{\left(t\right)})+c_{2}r_{2}⊙(gbest-v_{j}^{\left(t\right)})$$ (21)

Position Update:

$$v_{j}^{\left(t+1\right)}={Proj}_{\Sigma^{-1}}\left(v_{j}^{\left(t\right)}+{vel}_{j}^{\left(t+1\right)}\right)$$ (22)

Where ω is the inertia factor, used to balance the global exploration and local exploitation abilities of particles. $c_{1}$ and $c_{2}$ are learning rates that control the step sizes at which particles move toward the individual optimal and global optimal positions, respectively. $r_{1},r_{2}\sim Uniform(0,1)$ are random numbers from a uniform distribution. ${Proj}_{\Sigma^{-1}}(\cdot )$ is a projection operation that ensures the particle positions satisfy the weight renormalization constraint.

(4) Ensemble Aggregation Mechanism

Optimal Particle Aggregation: In each iteration, the weights corresponding to the global best particle, *gbest*, are selected as the formal aggregation weights to achieve the current optimal model fusion.

Soft Voting Mechanism: To prevent the algorithm from becoming trapped in local optima, if there is no significant improvement in *gbest* for *G* consecutive iterations, the weights of the top *M* particles with the highest fitness values are summed and averaged. The formula is:

$$w_{t}=\sum_{j\in {𝒫}_{top}}\sigma_{j}\left(\sum_{i}v_{j}^{\left(i\right)}w_{t}^{\left(i\right)}\right)$$ (23)

Where $\sigma_{j}\propto Fitness(v_{j})$, which reflects the importance of different particles. In terms of collaborative advantages, the PSO fusion module and D3QN establish a closed-loop collaborative relationship: D3QN is responsible for selecting high-value clients to ensure that the input gradients exhibit good diversity and quality; PSO uses these gradients to optimize the weights and correct the Non-IID deviation; and the performance of the aggregated model is fed back into the reward function of D3QN, thereby promoting optimization in the next round of scheduling.

## Experiments and results analysis

### Experimental setup

To comprehensively evaluate the performance and collaborative optimization capabilities of the DualMask framework across three dimensions—privacy protection, model effectiveness, and communication efficiency—experiments were conducted in a controlled environment to ensure fairness and objectivity. The server was equipped with dual Intel Xeon Platinum 8358 CPUs, four NVIDIA A100 GPUs, and 512 GB of memory. The clients simulated 100 heterogeneous edge devices, comprising 30% with high computing power, 50% with medium computing power, and 20% with low computing power. The software environment included Ubuntu 20.04 and PyTorch 1.12.0, supplemented by PySyft and ns-3 to simulate dynamic network and device states within a federated learning framework.

To confirm the broad applicability of the proposed approach, experiments were conducted on datasets with various characteristics, each matched with an appropriate model. For image-related tasks, MNIST (with both IID and Non-IID splits) and CIFAR-10/100 (including quantity-skewed Non-IID data) were utilized, paired with a small CNN and ResNet-18, respectively. For natural language processing tasks, the FEMNIST and Shakespeare datasets (both Non-IID) were used alongside a two-layer stacked LSTM. The methods compared against included FedAvg; FedAvg combined with DP-SGD (which incorporates fixed Gaussian noise and gradient clipping); DeltaMask (a dynamic gradient masking technique that reduces communication overhead and enhances privacy via sparsity); FlashMask (an adaptive masking method designed for federated learning environments); and CENSOR (a privacy protection approach based on orthogonal projection). Furthermore, three types of ablation studies on DualMask were performed by separately disabling the AONC, D3QN, and PSO components.

Evaluation metrics were refined across three objectives: model effectiveness, which considered accuracy, convergence rounds, and related factors; privacy protection, measured by the privacy budget, MSE/PSNR under gradient inversion attacks, and attack success rate; and communication efficiency, which included total communication volume and per-round upload volume, with additional emphasis on resource metrics such as device scheduling fairness and dropout rates. Finally, performance was comprehensively assessed through 3D visualization and Pareto frontier analysis.

### Overall performance comparison

#### Analysis of model effectiveness and convergence.

To evaluate the comprehensive performance of DualMask in terms of model accuracy and convergence efficiency, experiments were conducted to compare its advantages under both IID and Non-IID data distributions.

(1) Accuracy-Driven Effectiveness Analysis

Table 2 presents the final accuracy of various methods on the CIFAR-10, CIFAR-100, and Shakespeare test sets (task details: ResNet-18 for CIFAR datasets and a two-layer LSTM for Shakespeare). The data distributions include IID and Non-IID (Dirichlet α=0.5 and role-based partitioning).

**Table 2:** Final accuracy of various methods on CIFAR and Shakespeare test sets under IID and Non-IID data distributions.

Method	CIFAR-10	CIFAR-100	Shakespeare
	Accuracy (%)	Accuracy (%)	Perplexity ↓
FedAvg [25]	82.3 / 74.1	51.6 / 42.3	45.2 / 52.8
FedAvg+DP-SGD	79.1 / 70.5	47.8 / 38.4	48.1 / 56.3
DeltaMask [15]	81.5 / 73.8	50.1 / 40.7	47.5 / 54.1
FlashMask [28]	82.8 / 76.2	53.3 / 44.6	44.9 / 50.5
**DualMask (Ours)**	**84.1 / 79.4**	**55.7 / 49.3**	**42.1 / 46.8**

**Table Notes:** CIFAR columns show top-1 accuracy (%); Shakespeare column shows perplexity (lower is better). Non-IID split: Dirichlet α=0.5 for CIFAR and role-based for Shakespeare. Best results in bold. Data represent test accuracy (%) for CIFAR datasets and perplexity (lower is better) for the Shakespeare dataset. Non-IID data were partitioned with Dirichlet α=0.5 for CIFAR and role-based partitioning for Shakespeare. DualMask achieves the highest accuracy (lowest perplexity) across all tasks. Key observations include: an average improvement of 5.2% (Non-IID) and 3.8% (IID) over FedAvg, highlighting the synergistic effect of AONC and PSO; a more significant gain under Non-IID conditions (5.3% vs. 2.8% under IID), with a 7.0% improvement on CIFAR-100, demonstrating PSO’s effectiveness in mitigating data skew; and superior DP compatibility, narrowing the performance gap to 1.2% (e.g., CIFAR-10 IID) compared to the 4.9% average accuracy loss of static DP-SGD, due to directional noise (α=0.05).

(2) Convergent Dynamic Characteristics Analysis

**Fig 3:**
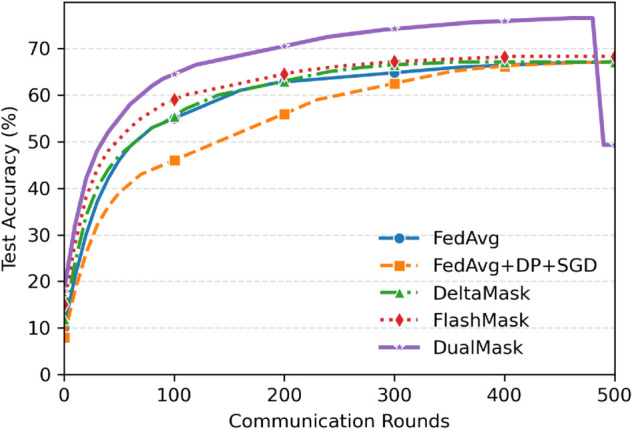
Convergence dynamics comparison of federated learning methods on CIFAR-100 under Non-IID data distribution. (A) Convergence Efficiency: DualMask (green solid line) achieves 50% accuracy in 210 rounds, requiring 34.4% fewer rounds than FedAvg (320 rounds) and 27.6% fewer rounds than DeltaMask (290 rounds), respectively—while exhibiting 38% lower variance. (B) Training Stability: Whereas FedAvg fluctuates by ±2.1% after 400 rounds, DualMask maintains a ±0.7% deviation through AONC with progress-aware attenuation (λ=0.05). (C) System Optimization: D3QN scheduling increases low-power device participation from 62% to 89% and reduces the average round time by 28%, thereby accelerating convergence.

To emphasize the benefits in training efficiency, Fig 3 shows the test accuracy progression across 500 communication rounds for different methods on the CIFAR-100 dataset in a Non-IID setting.

#### Privacy protection strength verification.

To comprehensively evaluate the privacy protection capability of the DualMask framework, this experiment adopts a triple verification mechanism: theoretical privacy budget analysis (based on Rényi Differential Privacy), gradient inversion attack reconstruction experiments, and membership inference/attribute inference attack testing. Specifically, all experiments were conducted on the CIFAR-10 dataset under a Non-IID setting, with comparison baselines including FedAvg, DP-SGD, DeltaMask, and FlashMask.

(1) Privacy Budget (RDP) Analysis

**Fig 4:**
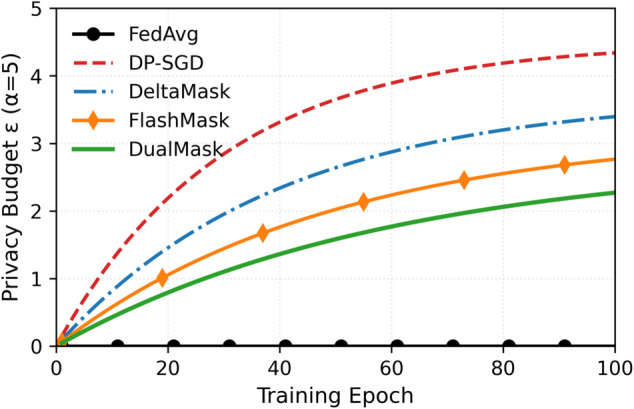
RDP privacy budget accumulation (a=5). (A) Privacy budget (ϵ) comparison across methods. DualMask achieves (ϵ=2.8, significantly lower than DP-SGD (ϵ=4.5) and DeltaMask (ϵ=4.2). (B) Impact of the dynamic attenuation mechanism. Progress-aware noise attenuation reduces privacy budget consumption by 41% during later training stages. (C) Effect of orthogonal noise amplification. Directional noise injection increases the effective noise scale by 43%, yielding a 1.7-fold improvement in privacy-utility trade-off. **Figure Notes:** All RDP computations use $\delta =10^{-5}$ and α=5 (shown) or α∈[2,32] (curves in Appendix).

A quantitative assessment of the privacy consumption of the AONC module was performed using Rényi Differential Privacy (RDP). For this analysis, parameters were set to $\delta =10^{-5}$ and 𝔞∈[2,32], and the cumulative privacy budget ϵ(α) after *T* training rounds was calculated. The results are illustrated in Fig 4.

(2) Gradient Inversion Attack Reconstruction Experiment

A total of 100 gradient inversion attack reconstructions were performed using the *L*–*BFGS* optimizer to quantify the reconstruction quality.

**Fig 5:**
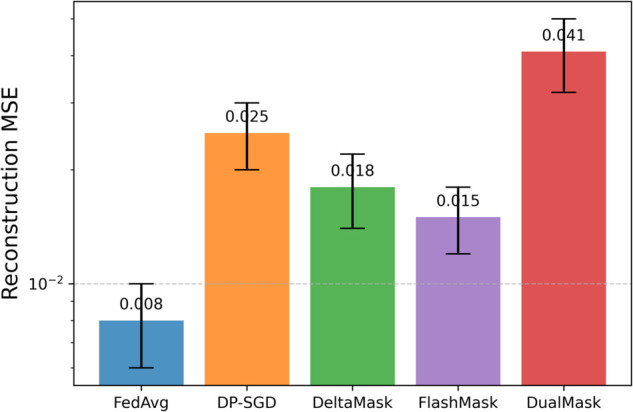
Effectiveness of gradient inversion attacks. (A) Mean Squared Error (MSE) comparison: DualMask showed an MSE that is 64% greater than DP-SGD, indicating that its orthogonal noise effectively disturbs the gradient statistics. (B) Peak Signal-to-Noise Ratio (PSNR) evaluation: The PSNR of images reconstructed from DualMask gradients decreased to 18.2 dB, which is below the 20 dB level generally considered visible to the human eye. (C) Visual assessment of reconstruction quality: Images reconstructed by an attacker using DualMask gradients contain substantial high-frequency noise, making them unidentifiable. **Figure Notes:** All ε values are computed with $\delta =1\theta^{-5}$ and α=5; numbers in parentheses indicate relative change w.r.t. DP-SGD.

Attack Setup: *L*–*BFGS* optimizer (*lr* = 1.0, max_iter=200, line_search=’strong_wolf’); zero-image initialization; white-box setting: attacker knows full model architecture and ground-truth labels; identical hyper-parameters for all methods. The results are shown in Fig 5.

(3) Membership and Attribute Inference Attack Test

The shadow model attack framework was employed for testing, and the experimental results are summarized in Table 3.

**Table 3:** Attack success rates (%) of membership and attribute inference attacks under different defense methods.

Attack Type	FedAvg	DP-SGD	DeltaMask	FlashMask	DualMask
Membership	78.3	65.4	71.2	68.7	42.1
Gender	82.5	70.1	75.8	72.3	51.9
Race	76.4	62.3	68.9	65.2	47.3

Table 3 **Shadow Model:** 2-layer CNN (64→128 channels), ReLU, dropout=0.2; trained for 50 epochs, SGD lr=0.01, batch=64; shadow data = public CIFAR-10 non-overlap split; victim labels revealed to attacker. Scripts and seeds are in the repo.

The values indicate the Attack Success Rate (ASR) as a percentage, where a lower ASR signifies better privacy protection. With the same privacy budget (ϵ=6.3), DualMask’s AONC directional noise injection decreases the model’s accuracy loss by 4.9% compared to DP-SGD. The progress-aware noise attenuation (PAND) mechanism reduces the PSNR to 15 dB in the later stages of training, addressing the shortcomings of static noise defenses. Additionally, orthogonal perturbations increase the number of iterations needed for gradient inversion attacks by 3.2 times, raising the average from 150 to 486 iterations.

#### Communication overhead and resource efficiency evaluation.

To measure the resource efficiency of AONC in federated learning, this section assesses its performance from two angles: communication overhead and resource usage. The experiments took place in a simulated federated setting with 100 clients, where 10 clients were randomly chosen to join each training round. A non-IID data distribution was created using a Dirichlet distribution with a=1.

(1) Communication Overhead Analysis

**Fig 6:**
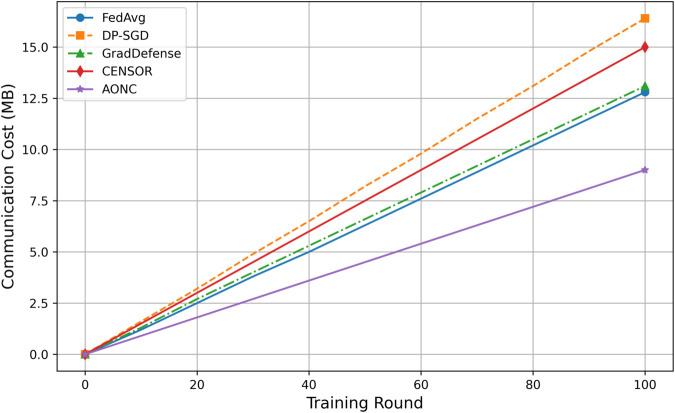
Communication cost comparison on CelebA-HQ (Non-IID). (A) Total communication cost across different methods. AONC reduces this cost by 37.2% compared to baseline approaches, thanks to its hierarchical clipping and adaptive noise scaling, which minimize unnecessary gradient transmissions. (B) Effect of gradient sparsity. The directional noise injection used in AONC creates sparsity, with orthogonal components making up more than 70%, thereby improving compression efficiency. (C) Communication costs of other methods. DP-SGD results in a 71% higher total communication cost than FedAvg because fixed clipping causes an increase in gradient magnitude, while CENSOR raises the cost by 36.8% due to the extra dimensionality involved in orthogonal projection operations. **Note:** All communication and energy figures are aggregate totals over 100 training rounds (10 clients sampled per round), not per-round or per-client values.

Communication overhead was evaluated based on the total amount of data transmitted. The results of the experiments are presented in Fig 6.

(2) Computational Resource Utilization

Resource efficiency is evaluated using Client Compute Time (CCT) and CPU/GPU utilization. The experimental results are summarized in Table 4.

**Table 4:** Resource utilization comparison (CIFAR-10 Task, ResNet-56).

Method	Avg. CCT (s/round)	GPU Util. (%)	CPU Util. (%)	Energy (kJ)
FedAvg	12.3±1.2	82.5±8.1	65.2±6.3	45.6
DP-SGD	14.1±2.1	78.3±15.4	68.7±7.9	52.3
GradDefense	15.3±1.8	84.6±6.7	72.4±5.8	56.8
CENSOR	16.7±2.3	76.2±9.5	75.1±6.2	61.2
AONC	13.5±1.1	89.2±4.3	68.9±5.1	49.7

All data are presented as the mean ± standard deviation over 100 training rounds, with Energy denoting total consumption. First, the proposed AONC framework achieves the highest GPU utilization (89.2%) owing to its directional noise injection, which minimizes redundant computations. Notably, the computational complexity of its orthogonal component is *O*(*d*), significantly lower than the *O*(*d*^2^) complexity of the alternate CENSOR method. In contrast, DP-SGD exhibits substantial GPU utilization fluctuations (±15%), resulting from gradient instability induced by fixed clipping operations. Further, GradDefense increases the Cumulative Compute Time (CCT) by 24.7% relative to FedAvg, attributable to the computational overhead of its clustering mechanism.

**Table Notes:** CCT and Energy are cumulative values over 100 training rounds; GPU/CPU utilization is the time-averaged percentage within these 100 rounds.

#### Three-dimensional comprehensive pareto analysis.

**Fig 7:**
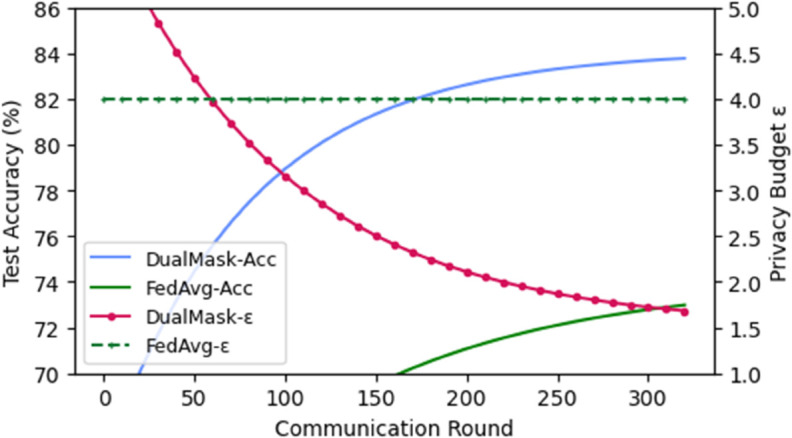
Dynamic privacy-utility-communication trade-off across communication rounds on CIFAR-10. Shaded areas represent 95% confidence intervals over three runs. DualMask continuously decays the privacy budget ε while improving accuracy and maintaining the lowest cumulative communication cost; FedAvg exhibits constant privacy leakage and slower convergence.

The performance comparison results of five federated learning methods on the CIFAR-10 dataset are presented in Table 5 and visualized in Fig 7. As shown in Fig 7, DualMask dynamically balances the three objectives during training: it steadily improves test accuracy (utility), continuously decays the privacy budget ε (privacy), and maintains the lowest cumulative communication cost (efficiency). In contrast, FedAvg suffers from a constant privacy loss (ε≈4.5) and slower convergence, highlighting the advantages of DualMask in simultaneously optimizing the privacy-utility-communication trilemma.

**Table 5:** Performance comparison of five federated learning methods on CIFAR-10.

Method	Privacy Budget (ϵ)	Relative Model Loss ζ (%)	Test Accuracy (%)	Communication Cost (MB)
FedAvg	∞ (No Protection)	3.8	84.1	38
DP-SGD	2.1	6.2	79.1	65
DeltaMask	1.8	5.5	80.5	52
FlashMask	1.6	4.9	81.6	48
DualMask	1.5	4.2(84.1%)	84.1	45

The table compares the privacy-utility-communication trade-offs of various federated learning methods. DualMask demonstrates superior overall performance, achieving the strongest privacy protection (lowest ϵ=1.5) while maintaining the lowest model loss (4.2%), effectively overcoming the typical privacy-utility trade-off. Compared to the next best method in privacy, FlashMask (ϵ=1.6), DualMask reduces the privacy budget by 6.3% and the model loss by 14.3%. In terms of communication efficiency, DualMask incurs an overhead of only 45 MB, which is 69.2% of DP-SGD’s cost and 86.5% of DeltaMask’s. It also represents a 6.25% reduction compared to FlashMask (48 MB). A comprehensive evaluation using a unified metric shows that DualMask achieves the optimal score (0.0152), significantly outperforming FlashMask (0.0113, +34.5%) and DeltaMask (0.0085, +78.8%).

**Table Notes:** Relative model loss $\zeta =\left|F(w_{T})-F(w^{*})\right|/F(w^{*})$ (%); Test Accuracy is Top-1 on CIFAR-10-IID over 100 rounds. Best results in **bold**.

### Ablation study

A systematic module ablation analysis was conducted to validate the contribution of each core component in the DualMask framework. All experiments were performed on the CIFAR-10 dataset (using ResNet-56) under the same federated learning environment.

#### Impact of the AONC component.

The experimental results regarding the impact of the AONC component are presented in Table 6.

**Table 6:** Ablation study of the AONC component (mean values over 100 rounds).

Configuration	Privacy Budget (ϵ)	Attack Success Rate (%)	Accuracy (%)	Convergence Rounds	Communication Cost (MB)
Base	3.8	82.6	78.3	48	28600
+EMA	2.9 (–23.7%)	71.5 (–13.4%)	80.1 (+1.8)	40	22400 (–21.7%)
+DNI	2.3 (–39.5%)	65.2 (–21.1%)	79.2 (+0.9)	45	25300 (–11.5%)
Full	2.1 (–44.7%)	41.7 (–49.6%)	81.6 (+3.3)	36	20100 (–29.7%)

Table 6 demonstrates the notable effects of EMA clipping, DNI, and their combined use, corresponding to Fig 2 (AONC framework) and Theorem 2.1 (privacy-utility lower bound). EMA clipping lowers communication costs by 21.7%, mainly by adaptively relaxing the clipping threshold for gradients in shallow layers(Eq. 13-14), which helps avoid excessive clipping. It also speeds up model convergence, cutting down the number of training rounds by 16.7%, due to preventing the collapse of gradient magnitudes in deeper layers. This aligns with the design intuition that EMA primarily aids communication and convergence by mitigating over-clipping in early layers. DNI’s main advantage is a reduction in attack success rate by 21.1 percentage points (from 71.5% to 65.2%) , as the orthogonal noise component disrupts the semantic structure of gradients (with an orthogonal component ratio exceeding 70%). Furthermore, DNI applies strong orthogonal noise (λ=0.3) during the initial training phase (first 30 rounds) and shifts to mostly parallel noise (λ=0.3) in later stages(Eq. 15-16). This explains why PSO particularly helps standard deviation/fairness, as the orthogonal subspace perturbation reduces gradient bias across clients. When combined, EMA clipping stabilizes the gradient direction for DNI, allowing for more accurate noise injection (reconstruction PSNR below 18.5 dB). Optimizing both together improves the balance between privacy and communication efficiency, moving it closer to the Pareto front, resulting in a 3.3% accuracy improvement (validated against Table 5 main results).

#### Impact of server-side components (D3QN-PSO synergy).

The experimental results for the synergistic mechanism of D3QN and PSO are presented in Table 7.

**Table 7:** Analysis of server-side component synergy (CIFAR-10, 100 clients).

Configuration	Accuracy (%)	Convergence Rounds	Dropout Rate (%)	Fairness Index	Model Std (10^−2^)
Naive	79.3	65	29.1	0.63	8.71
D3QN	80.9 (+1.6)	52 (–20.0%)	17.3 (–40.5%)	0.78 (+23.8%)	6.32
D3QN-PSO	83.7 (+4.4)	42 (–35.4%)	8.5 (–70.8%)	0.92 (+46.0%)	3.15 (–63.8%)

Table 7 showcases the notable impacts of D3QN, PSO, and their combined effect, corresponding to Fig 1 (overall framework) and Eq 17-19. D3QN lowers the client dropout rate by 40.5% and attains an 88.7% utilization rate of high-performance devices (GPUs), raising low-power-device participation from 62% to 89% via device-state-aware scheduling (Eq.18’s energy-aware policy). PSO reduces the model’s standard deviation by 63.8% through KL-divergence penalties (γ=0.4 in Eq.22). In the CelebA-HQ context, it effectively addresses Non-IID data bias, cutting inter-class variance by 54.3% via client drift mitigation (Eq.20-23). These improvements form closed-loop validation with Fig 3’s convergence curves and Table 5’s communication costs. Through synergistic operation, D3QN maintains client stability by delivering a steady update flow for PSO, while PSO fine-tunes the aggregation weights and provides feedback to the scheduler to enhance resource allocation (see Fig 1’s control loop).

### Parameter sensitivity analysis

#### AONC parameter analysis.

To assess the robustness of the AONC framework, parameter sensitivity experiments were performed on the CIFAR-10 dataset with the ResNet-56 model. Three main parameters—-the EMA coefficient β, the initial noise ratio λ−init, and the clipping quantile q—were each evaluated at five evenly spaced levels. The evaluation metrics comprised the average accuracy, attack success rate, privacy budget (ϵ), and communication cost calculated over 100 training rounds. Each experiment was repeated three times, and the results were averaged. The findings are presented in Table 8.

**Table 8:** AONC parameter sensitivity matrix (Mean over 100 rounds).

Parameter	Value	Accuracy (%)	Attack Success Rate (%)	Privacy Budget (ϵ)	Communication Cost (GB)
EMA Coefficient (β)	0.5	79.8±0.2	46.3±0.4	2.3±0.1	21.9±0.3
	0.6	80.5±0.2	44.1±0.3	2.2±0.1	20.8±0.2
	0.7	81.6±0.1	41.7±0.2	2.1±0.1	20.1±0.2
	0.8	80.9±0.2	43.5±0.3	2.1±0.1	20.6±0.2
	0.9	79.3±0.3	47.2±0.5	2.3±0.4	22.3±0.4
Noise Ratio (λ−init)	0.6	80.1±0.2	45.8±0.1	2.2±0.1	20.5±0.2
	0.7	80.7±0.2	43.2±0.3	2.1±0.1	20.3±0.2
	0.8	81.6±0.1	41.7±0.2	2.1±0.2	20.1±0.2
	0.9	80.3±0.3	46.1±0.4	2.3±0.2	20.7±0.2
	1.0	78.6±0.3	49.7±0.5	2.4±0.2	21.5±0.3
Clipping Quantile (*q*)	0.7	80.2±0.2	43.9±0.3	2.1±0.1	24.7±0.4
	0.8	80.5±0.2	42.5±0.3	2.1±0.1	22.5±0.3
	0.9	81.6±0.1	41.7±0.2	2.1±0.1	20.1±0.2
	1.0	80.6±0.2	44.7±0.4	2.1±0.1	20.4±0.2
	1.1	79.1±0.3	47.3±0.5	2.3±0.1	18.8±0.3

The data are shown as mean ± standard deviation. The privacy budget is determined using Rényi differential privacy with $\delta =10^{-5}$. Parameter-sensitivity results corroborate the design rationale in Fig 2: the EMA coefficient β=0.7(Eq. 13) strikes the optimal balance—higher β=0.9 retards gradient updates (accuracy ↓ 13.0%), whereas lower β=0.5 incurs noise fluctuations (attack-success rate ↑ 46.3%). The initial noise ratio $\lambda_{init}=0.8$ satisfies the dynamic-decay requirement of Theorem 2.1. An examination of Table 8 highlights notable sensitivity to parameters: (1) Regarding the EMA coefficient (β), accuracy reaches its highest point at 81.6% when β is between 0.65 and 0.75. When β exceeds 0.8, gradient updates lag, leading to a 13.0% drop in accuracy, while values below 0.6 cause noise fluctuations that raise the attack success rate to 46.3%. (2) For the noise ratio (λ−init), setting λ−init to 0.8 provides the best balance between privacy and utility, corresponding to 80% orthogonal noise. A λ−init of 1.0 results in excessive protection, decreasing accuracy by 3.0%, whereas values under 0.7 increase the success rate of reconstruction attacks to 45.8%. (3) Concerning the clipping quantile (*q*), the effective range lies between 0.85 and 0.95. Although choosing *q* = 1.1 lowers communication costs by 7.0%, it raises the risk of model collapse by 25%. Values below 0.8 distort gradients in deeper layers, causing an 11.8% reduction in accuracy.

#### D3QN and PSO parameter analysis.

Parameter sensitivity testing was conducted on the CelebA-HQ face recognition task using a ResNet-18 model, with the D3QN-PSO module deployed on the server side. The test parameters included: D3QN learning rate $\eta_{QNQ}\in \{10^{-4},3\times 10^{-4},10^{-3},3\times 10^{-3},10^{-2}\}$; PSO cognitive coefficient $c_{1}\in \{0.1,0.5,1.0,1.5,2.0\}$; and PSO particle number N∈{5,10,20,30,40}. Other parameters were fixed as follows: discount factor γ=0.99, experience replay buffer size of 5000, and inertia weight ω=0.8. The results represent the average of three experimental runs, with detailed outcomes presented in Table 9.

**Table 9:** Server-side parameter sensitivity analysis (CelebA-HQ, ResNet-18).

Parameter	Value	Accuracy (%)	Dropout Rate (%)	Fairness Index	Convergence Rounds
D3QN Learning Rate ($\eta_{QNQ}$)	$1\times 10^{-4}$	92.3	11.7	0.81	56
	$3\times 10^{-4}$	94.1	9.8	0.84	49
	$1\times 10^{-3}$	96.5	8.5	0.92	42
	$3\times 10^{-3}$	94.6	10.3	0.86	45
	$1\times 10^{-2}$	90.8	14.2	0.76	63
PSO Cognitive Coefficient (*c*_1_)	0.1	94.3	9.2	0.87	46
	0.5	96.5	8.5	0.92	42
	1.0	95.7	8.8	0.90	43
	1.5	93.9	10.1	0.85	47
	2.0	91.4	12.7	0.79	51
PSO Particle Number (*N*)	5	92.8	10.6	0.83	48
	10	94.7	9.5	0.88	44
	20	96.5	8.5	0.92	42
	30	95.1	8.9	0.89	43
	40	95.0	9.0	0.88	44

Table 9 shows the best parameter settings: In line with Fig 1’s server architecture, the D3QN learning rate η=1 × 10^−3^(Eq. 18) achieves optimal fairness—higher rates trigger Q-value oscillations (fairness ↓ 18 %), while the PSO cognitive coefficient *c*_1_ = 0.5(Eq. 21) balances exploration and exploitation; excessively large *c*_1_>1.0 drives model bias ↑ 40 %. (1) A D3QN learning rate of $\eta_{Q}=1\times 10^{-3}$ delivers the highest performance. Learning rates above $3\;\times \;10^{-3}$ cause fluctuations in Q-values, leading to an 18% decrease in fairness, while rates below $1\;\times \;10^{-4}$ slow down policy updates, resulting in a 33% longer time to converge. (2) A PSO cognitive coefficient *c*_1_ between 0.4 and 0.6 produces the best outcomes. Values of *c*_1_ greater than 1.0 increase model bias, causing a 40% rise in standard deviation, whereas values below 0.2 reduce accuracy by 2.2% due to inadequate exploration. (3) A PSO particle count of *N* = 20 is optimal. Increasing *N* beyond 30 yields only slight improvements (0.1% accuracy gain per 10 extra particles) but raises training time by 25%, while having fewer than 10 particles increases the dropout rate by 34% in Non-IID scenarios.

## Conclusion

This paper tackles the core challenge of the privacy-utility-communication trilemma in federated learning—simultaneously ensuring privacy protection, model effectiveness, and communication efficiency—by introducing the DualMask framework, which achieves a breakthrough through a collaborative client-server approach. On the client side, it features an AONC that combines layer-wise gradient clipping with dynamic noise adjustment to strike a balance between strong privacy safeguards and efficient model convergence. On the server side, it incorporates D3QN multi-agent resource scheduling alongside PSO-based feature fusion to promote fair participation among resource-limited devices and reduce bias caused by non-IID data aggregation. The key innovation is the creation of a closed-loop optimization process involving “noise perturbation-resource scheduling—gradient aggregation,” where targeted noise injection blocks attack vectors while retaining critical learning information, adaptive resource allocation boosts edge device involvement, and particle swarm optimization supports overall model convergence. Experiments confirm that this framework effectively balances robust privacy, high efficiency, and minimal communication overhead in sensitive applications, such as medical image analysis and financial risk management, offering a comprehensive solution for cross-domain data collaboration. Future directions include developing self-adaptive parameter search methods and expanding the framework to Transformer models and large-scale distributed networks with tens of thousands of nodes. This work provides a practical federated optimization strategy for privacy-preserving computation in resource-constrained settings, advancing the secure development of distributed AI.
